# Solid-state fermentation of hemp waste: enhancing the performance of *Hermetia illucens* larvae and altering the composition of hemp secondary metabolites

**DOI:** 10.3389/fbioe.2025.1449233

**Published:** 2025-01-24

**Authors:** Wael Yakti, Nadja Förster, Marcus Müller, Sebastian Beck, Simon Schulz, Inga Mewis, Christian Ulrichs

**Affiliations:** ^1^ Faculty of Life Sciences, Thaer-Institute of Agricultural and Horticultural Sciences, Urban Plant Ecophysiology Division, Humboldt-Universität zu Berlin, Berlin, Germany; ^2^ Department of Chemistry, Faculty of Mathematics and Natural Sciences, Applied Analytical and Environmental Chemistry, Humboldt-Universität zu Berlin, Berlin, Germany

**Keywords:** black soldier fly, cannabis, plant secondary metabolites, solid-state fermentation, insect protein

## Abstract

Solid-state fermentation (SSF) can increase the nutritional quality of low value substrates for insects. In this study, SSF using different fungal species was applied on a hemp waste substrate, and the fermentation was followed by a black soldier fly larvae (BSFL) feeding experiment during which 300 larvae were grown on 200 g (20.1% DM) substrate for 7–9 days depending on the treatment. Besides assessing the BSFL performance parameters, the presence of hemp cannabinoids, flavonoids, and terpenes was assessed through the process and compared among the treatments. The results show that BSFL growth parameters varied depending on the fungal species used. Fermenting the substrate with *Ganoderma lucidum* can lead to an increase in the BSFL dry yield (4.54 g) compared to the untreated substrate (2.86 g), likely due to enhancing carbon accessibility in the substrate. SSF using *Trichoderma reesei* increased the cannabidiol and ∆9-tetrahydrocannabinol mass fractions in the substrate, and consequently in the produced BSFL biomass, while decreasing the amounts of acidic cannabinoids. Both *Hypsizygus ulmarius* and *Pleurotus ostreatus* effectively removed cannabinoids from the substrate. This study confirms that pre-treating hemp wastes via SSF can enhance their nutritional value and/or reduce bioactive secondary metabolites, with different fungal species offering different and complementary performances in achieving different biotechnological goals.

## 1 Introduction

Hemp (*Cannabis sativa* L.) cultivation has been gaining momentum globally due to its multiple uses, such as the production of fibers (Sankari, 2000), seeds (Xu et al., 2022) and flowers (Carus and Sarmento, 2016). The recent reforms in cannabis regulations in several counties have sparked rapid expansion within the hemp industry, leading to the introduction of numerous innovative products in the market (Crini et al., 2020). The plant is well known for the production of bioactive secondary metabolites, such as ∆9-tetrahydrocannabinol (∆^9^-THC) and cannabidiol (CBD). In the European Union (EU), the cultivation of industrial hemp with a maximum Δ^9^-THC content of 0.3% is permitted since 2021 (Union, 2021). Certified cultivars have low ∆^9^-THC content and predominantly contain CBD, which is now widely used in the treatment of various psychiatric and neurological disorders (Rock et al., 2018; Fiani et al., 2020). In many cases, industrial hemp is cultivated on a large scale for a specific purpose (e.g., seeds or flower production), resulting in the production of plant biomass that is often regarded as waste or a low-value by-product from the non-targeted parts of the plant in these cases. Such waste can have a high nutritional value for animals (Kleinhenz et al., 2020; Ely and Fike, 2022; Addo et al., 2023), but it also raises safety concerns as bioactive substances such as ∆^9^-THC can be found in the end animal products (Kleinhenz et al., 2020; Wagner et al., 2022). Using hemp-derived material as animal feed is prohibited in the United States (Gottlieb et al., 2018) while in the EU the use is restricted to seeds and seed press-cake (European Commission, 2009). This excludes the widely abundant vegetative plant parts and leads to hemp wastes ending up in landfill or compost. The efficient and safe utilisation of such residues holds great potential in supporting bio-economy visions and the transformation from linear to circular production systems (Ubando et al., 2020).

The use of insects in waste management is emerging as an innovative approach to convert a wide variety of organic waste streams to a high-value insect biomass (Kee et al., 2023; Yakti et al., 2023c). The saprophagous larvae of the black soldier fly (BSFL), *Hermetia illucens*, for instance, have been shown to thrive on different organic wastes (Kee et al., 2023). These larvae present a valuable biomass rich in protein and fats, making them a high-quality feed source (Raksasat et al., 2020; Siddiqui et al., 2022). A recent study has assessed the growth of BSFL on diets containing hemp wastes, revealing a potential utilisation of this waste stream without detecting ∆^9^-THC in the produced larvae (Yakti et al., 2023a), thus ensuring a safe employment in feed applications.

Hemp wastes are known to be rich in fibers (Carus and Sarmento, 2016), which, despite improving the physical properties of the feeding substrates (Grossule and Lavagnolo, 2020; Yakti et al., 2023b), are mostly indigestible and reduce the bioprocess efficiency (Peguero et al., 2021). The nutritional value of lignocellulosic substrates can be improved through solid-state fermentation (SSF) (Peguero et al., 2021). The process employs microbial strains that can produce a variety of enzymes such as cellulases, pectinases, and xylanases (Marzo et al., 2019; Vandenberghe et al., 2021). This has been shown in the case of hemp wastes, as the fermentation with *Pleurotus ostreatus* reduced the lignin in the substrate and enhanced the protein content (Eliopoulos et al., 2022). In the case of BSFL feeding substrates, *Trichoderma reesei* has been used to ferment banana peels prior to larvae feeding and this has led to improved larval biomass and bioconversion rate (Isibika et al., 2019). The use of SSF to enhance nutrient accessibility for BSFL across different substrates has the potential to elevate BSFL production, while harnessing the full capacity of agricultural waste resources. In the presented study, we hypothesise that SSF of hemp wastes using different fungal species will enhance the nutritional value of the substrate, thereby improving the performance and nutritional composition of BSFL. We also hypothesise that the SSF process will alter the secondary plant metabolites (cannabinoids, terpenes, and flavonoids) in hemp wastes and, subsequently, the produced BSFL. To test the hypothesis, hemp wastes underwent SSF with the fungi *P. ostreatus*, *Hypsizygus ulmarius*, *Ganoderma lucidum*, and *T. reesei,* and the resulting products were fed to BSFL. The effect of the SSF process on hemp secondary metabolites was also assessed and analysed in the fermented substrates and produced larvae.

## 2 Materials and methods

### 2.1 Hemp waste material

The hemp waste was provided by the company Die Hanflinge (Gumtow, Brandenburg, Germany) and comprises air-dried low-quality buds that were excluded from tea production. The waste stream also includes secondary stems along with leaves. The material was dried again at 60°C and ground to achieve <2 mm particle size.

### 2.2 Fungal strain selection and screening

Nineteen fungal strains were screened for their ability to grow on the hemp waste substrate in a Petri dish assay in order to identify candidate for the SSF. The strains screened were *P. ostreatus* MG1005*, P. ostreatus* var*. Florida* MG1015*, P. ostreatus* var*. Columbinus* MG1010*, Pleurotus eryngii* MG1105*, Pleurotus citrinopileatus* MG1205*, Pleurotus pulmonarius* MG1305*, Pleurotus djamor* MG1405*, H. ulmarius* MG1505*, Pleurotus eunosmus* MG1107*, Flammulina velutipes* MG4100*, Ganoderma applanatum* MG11600, *G. lucidum* MG11500, *Hericium coralloides* MG5510, *Hericium erinaceus* MG5500, *Hypholoma capnoides* MG1589, *Lentinula edodes* MG2500, *Stropharia rugosoannulata* MG2351, *T. reesei* DSM 768, and *Agaricus bisporus* ABW93. All strains except *T. reesei* and *A. bisporus* were obtained from MycoGenetics Pilz-Shop (Everswinkel, North Rhine-Westphalia, Germany), and are known to produce edible fruit bodies. The *T. reesei* strain was obtained from the Leibniz Institute DSMZ - German Collection of Microorganisms and Cell Cultures (Braunschweig, Lower Saxony, Germany). The *A. bisporus* strain was obtained from Pilzmännchen GbR (Malschwitz, Saxony Germany) All strains were maintained on PDA medium (Carl Roth, Karlsruhe, Baden-Wuerttemberg, Germany) until used in the assay.

A hemp-waste medium was prepared using 40 g/L pulverised hemp wastes and 15 g/L agar (Carl Roth, Karlsruhe, Baden-Wuerttemberg, Germany). The mixture was autoclaved and 15 mL were poured onto 9 cm Ø Petri dishes after homogenisation. 5 mm diameter plugs of growing mycelia on PDA were placed on the side of the Petri dishes and were allowed to grow for 12 days at 26°C. The fungal growth towards the other side of the medium was measured at days 3, 5, 7, 10, and 12, and the radial growth rate (mm^.^day^−1^) was calculated by dividing the total growth distance by the number of days needed to reach a distance of 6 mm. For species with low growth rate, the maximum growth distance (under 6 mm) was divided by 12 as the last day of measurements.

### 2.3 Solid-state fermentation of hemp waste

The dried hemp waste was mixed with distilled water to achieve a dry matter content of 26.8% (w/w). The mixture was stirred with a kitchen spatula and subsequently left to sit at room temperature for 1 hour. This enabled the formation of a wet porous substrate without the presence of excess free water. The substrate was then distributed to autoclavable containers (6 cm × 6 cm × 10 cm) each of which was filled with 150 g (volume of 280 mL). The containers were autoclaved at 121°C for 20 min and inoculated with *P. ostreatus* var. *Florida* MG1015 (PO), *H. ulmarius* MG1505 (HU), *G. lucidum* MG11500 (GL), and *T. reesei* DSM 768 (TR) by placing 5 plugs (5 mm diameter) of growing mycelia on the top of the hemp substrate. Plugs of PDA medium were used as a control and each treatment comprised 8 replicates (n = 8). The inoculated containers were incubated at 26°C until fungal mycelia had covered the bottom of the containers. Given the different growth rates of the fungal species, the treatments fully colonised by fungal mycelia were stored at 2°C until the rest of the treatments were also fully colonised. Hence, TR containers were transferred to 2°C on day 12, PO at day 14, GL at day 19, while HU needed 21 days to fully cover the substrate. On the 21st day, the substrates were taken out of the containers and samples were collected from 3 replicates per treatment (n = 3). The samples were stored at −80°C for later analyses of secondary plant metabolites.

### 2.4 BSFL feeding experiment

Newly hatched black soldier fly neonates obtained from FreezeM and Hermetia Deutschland GmbH and Co. KG (Baruth/Mark, Brandenburg, Germany) were used in the BSFL feeding experiment. The neonates received GoldDott grains-based chicken feed (Agravis Raiffeisen AG, Velten, Brandenburg, Germany) and were incubated at 30°C to reach an average weight of 19 mg on day 7 (on which the experiment started). The BSFL received PO-, HU-, GL-, TR-fermented substrates, or the substrate inoculated with sterile PDA plugs as control (K2). An additional control treatment with the initial untreated hemp waste was also added (K1).

For the preparation of the feeding substrates for the BSFL, the replicates of each SSF treatment were pooled, homogenised, and the dry matter content (DMC) was determined by drying samples of 100 g at 80°C until no further weight reduction was observed. The DMC of all substrates was adjusted to that of the TR treatment, which had the lowest DMC (20.1%) by adding distilled water. The untreated control substrate was prepared by mixing distilled water with the initial dry hemp waste substrate to achieve 20.1% DMC. 200 g of DMC-adjusted substrates were put in plastic rearing boxes (10.5 cm Ø and 7 cm high) and 300 BSFL were added on the top of each of the substrates. The experiment had 6 treatments and 5 replicates (n = 5). The rearing boxes had top openings (7.5 cm Ø) sealed with 1 mm mesh. The BSFL were grown at 28°C and the growth was assessed by collecting and weighing at least 30 individuals on days 2, 4, 7, 8, and 9. The BSFL were harvested earlier than day 9, when at least 3 out of the 5 replicates exhibited weight loss. The BSFL were separated from the remaining substrates, counted, and weighted before and after freeze-drying. The feed conversion ratio (FCR) was determined on a dry matter basis by dividing the reduction in dry substrate by the dry weight gain of the BSFL.

### 2.5 Elemental analyses and crude protein quantification

The start hemp waste, the fermented and the autoclaved substrates, as well as the produced larvae were lyophilised and stored at −80°C until further processing. The BSFL were manually blended with a mortar and pestle in liquid nitrogen and the substrates were processed into powders using a Retsch MM 400 swing mill (Retsch GmbH, Haan, North Rhine-Westphalia Germany). The nitrogen (N) and carbon (C) contents were determined in the produced larvae (n = 3–5) and in the initial hemp waste according to LUFA Bd. III, 4.1.2., and the crude protein was calculated based on the conversion factor of 4.43 (Smets et al., 2021). Phosphorus (P), Calcium (Ca), Magnesium (Mg), Copper (Cu), Potassium (K), Iron (Fe), Magnesium (Mg), Manganese (Mn), Sulfur (S) and Zinc (Zn) were analysed in the initial hemp substrate and the produced larvae using ICP-OES (DIN EN ISO 11885) as previously described in Yakti et al. (2022). Due to the insufficient amount of BSFL biomass produced in the scale of the experiment, the larvae of all replicates were pooled into one sample and analysed accordingly for all mentioned elements other than N and C.

### 2.6 Hemp secondary metabolites

Hemp cannabinoids, flavonoids, and terpenes were analysed in the initial hemp waste, the fermented substrates (n = 3), and in the produced larvae (n = 5). The mass fractions of cannabinoids and flavonoids were determined via high pressure liquid chromatography (HPLC). HPLC components (Autosampler, Pump, thermally-regulated column department, Photodiode Array Detector) including the software (Chromeleon 7.2) were supplied by ThermoFisher (ThermoScientific, Dreieich, Germany). The samples were analysed for cannabinoid contents according to the modified method of Mandrioli et al. (2019), described in Yakti et al. (2023a) and for flavonoids according to a method described by Förster et al. (2023).

For cannabinoid determination, 20 mg of lyophilised, pulverised substrate material (100 mg for BSFL material) were extracted with 750 µL of extraction solution (methanol/chloroform 9/1, v/v) for 10 min at room temperature and 500 rpm on a shaker (Eppendorf SE, Hamburg, Germany). The samples were centrifuged (6,800 × g, 5 min, room temperature) and the supernatant was collected in a glass vial. Thereafter, the pellet was re-extracted with 500 µL extraction solution twice. The combined supernatants were concentrated under a nitrogen stream to dryness and refilled with 500 µL 100% acetonitrile. The extract was filtered using 0.22 µm SpinX tubes (Costar, Corning, New York, NY, United States), filled in HPLC vials, and stored at −20°C until HPLC analysis. The extracts (10 µL) were analysed on an AcclaimTM RP18 column (3 μm, 120 Å, 2.1 × 250 mm, ThermoScientific) with a flow rate of 0.4 mL/min at a column temperature of 35°C at 265 nm using the eluents (A) 0.85% formic acid in ultrapure water and (B) 0.85% formic acid in 100% acetonitrile. The following gradient program was used: 70% B (0–3 min), 70%–85% B (3–10 min), 85%–95% B (10–17 min), 95%–100% B (17–18 min), and 100%–70% B (18–28 min). Commercially available standards of single compounds were used as references: cannabidiolic acid (CBDA), cannabigerolic acid (CBGA), cannabigerol (CBG), cannabidiol (CBD), ∆9-tetrahydrocannabinol (∆^9^-THC), cannabichromene (CBC), ∆9-tetrahydrocannabinolic acid (∆^9^-THC-A), and cannabichromenic acid (CBGA).

For flavonoid determination, 20 mg of lyophilised, pulverised substrate material (100 mg for insect material) were extracted with 300 µL 70% methanol (pH 4, acetic acid) for 15 min in ice water using sonification (Bandelin Sonorex, BANDELIN electronic GmbH and Co. KG, Germany). The pellet was re-extracted twice with 300 μL of the extraction solvent for 10 min. After each extraction step the samples were centrifuged for 5 min at 6,800 × g at 4°C and the supernatants were combined. Supernatants were concentrated (vacuum concentrator, ThermoScientific Savent SPD111V Concentrator, vacuum pump: Vacuumbrand PC 3001 series, CVC3000, Germany) to near dryness, dissolved in 50% methanol, and reconstituted to 1 mL. The extract was filtered using 0.22 µm SpinX tubes (Costar, Corning, New York, NY, United States), transferred to HPLC vials, and stored at −20°C until HPLC analysis. The extracts (10 µL) were analysed on an AcclaimPA (3 μm, 120 Å, 2.1 × 150 mm, ThermoScientific) protected by a pre-column (5 μm, 120 Å, 2 × 10 mm, ThermoScientific) with a flow rate of 0.4 mL/min at a column temperature of 35°C at 290 nm using the eluents (A) H_2_O (0.5% formic acid) and (B) 40% acetonitrile. The following gradient program was used: 0–1 min: 0.5% B, 1–10 min: 0.5%–40% B, 10–12 min: 40% B, 12–18 min: 40%–80% B, 18–20 min: 80% B, 20–24 min: 80%–100% B, 24–30 min: 100% B, 30–34 min: 100%–0.5% B, and 34–39 min 0.5% B). Quantification of single flavonoids was carried out against the internal standard 4-methoxycinnamic acid (1 mM, Sigma Aldrich, Germany). Commercially available standards of single compounds were used as reference (apigenin-7-glucoside, luteolin-7-glucoside, luteolin-7-glucoronide). Relative response factors of compounds with a similar chemical structure were used to correct for absorbance difference.

The identification of cannabinoids and flavonoids was based on their retention times and specific UV-spectra (if specific standards are commercially available), as well as mass spectrometry. MS/MS was performed by electrospray ionisation (ESI) on a ThermoScientific LXQ ESI-Ion Trap mass spectrometer (cannabinoids: negative and positive ion mode; flavonoids: negative mode). Mass spectra were recorded in the range from m/z 50 to 1,000. Instrument control and data processing were performed with Thermo Xcalibur Version 2.2 SP1.48.

Terpenes were extracted by adding 500 µL isooctane to 100 mg lyophilised and pulverised material. After sonification for 10 min in ice water (Bandelin Sonorex, BANDELIN electronic GmbH and Co. KG, Germany) and centrifugation (5 min at 6,800 × g at 4°C), the supernatant was collected in a glass vial. The pellet was re-extracted twice with 250 μL isooctane. The combined supernatants were concentrated under nitrogen stream to dryness and reconstituted with 300 µL isooctane, transferred to glass vials, and stored at −20°C until analysis. Terpenes were assessed via gas chromatography-mass spectrometry (GC-MS) as previously described in Beck (2022). In detail, terpenes were identified GC-MS on an Agilent 7890 GC system equipped with an Agilent HP5-MS ultra-inert column (30 m length, 0.25 mm i. d., 0.25 μm film thickness). A volume of 2 μL of sample was injected splitless, and separation was achieved using a helium flow of 2 mL ·min^−1^ and the following temperature gradient: 45°C for 5 min, 8°C·min^−1^ to 200°C, 200°C for 10 min. Eluting signals were detected via an Agilent 7076 MSD using an electron impact (EI) ionisation source and 70 eV ionization voltage, while the EI source was maintained at 230°C. Signals were acquired from m/z 50 to 500 at a scan speed of 1,562 u·s^−1^ and subjected to National Institute of Standards and Technology (NIST) database searches (NIST-2017) using NIST MS Search 2.3. The identity of identified terpenes was subsequently validated by using standard solutions (100 ng·mL^−1^) of respective terpenes in isooctane. Neophytadiene was purchased as analytical standard from Supelco (Bellefonte, PA, United States), caryophyllene from Toronto Research Chemicals (North York, Ontario, Canada) and isooctane (GC-MS grade) from Carl Roth (Karlsruhe, Germany).

### 2.7 Statistical analyses

Kruskal–Wallis test followed by Dunn-Bonferroni test was carried out on the data obtained from the screening of fungi “radial growth rate” and the data obtained from the analysis of cannabinoids as these parameters did not meet the assumptions of parametric tests. The BSFL growth parameters underwent one-way ANOVA followed by subsequent Bonferroni’s *post hoc* comparisons after verifying data normality and variance homogeneity. All data were analysed using SPSS version 28.0.0.0 (IBM Corp, Armonk, United States).

## 3 Results

### 3.1 Fungal strain selection and screening

**FIGURE 1:**
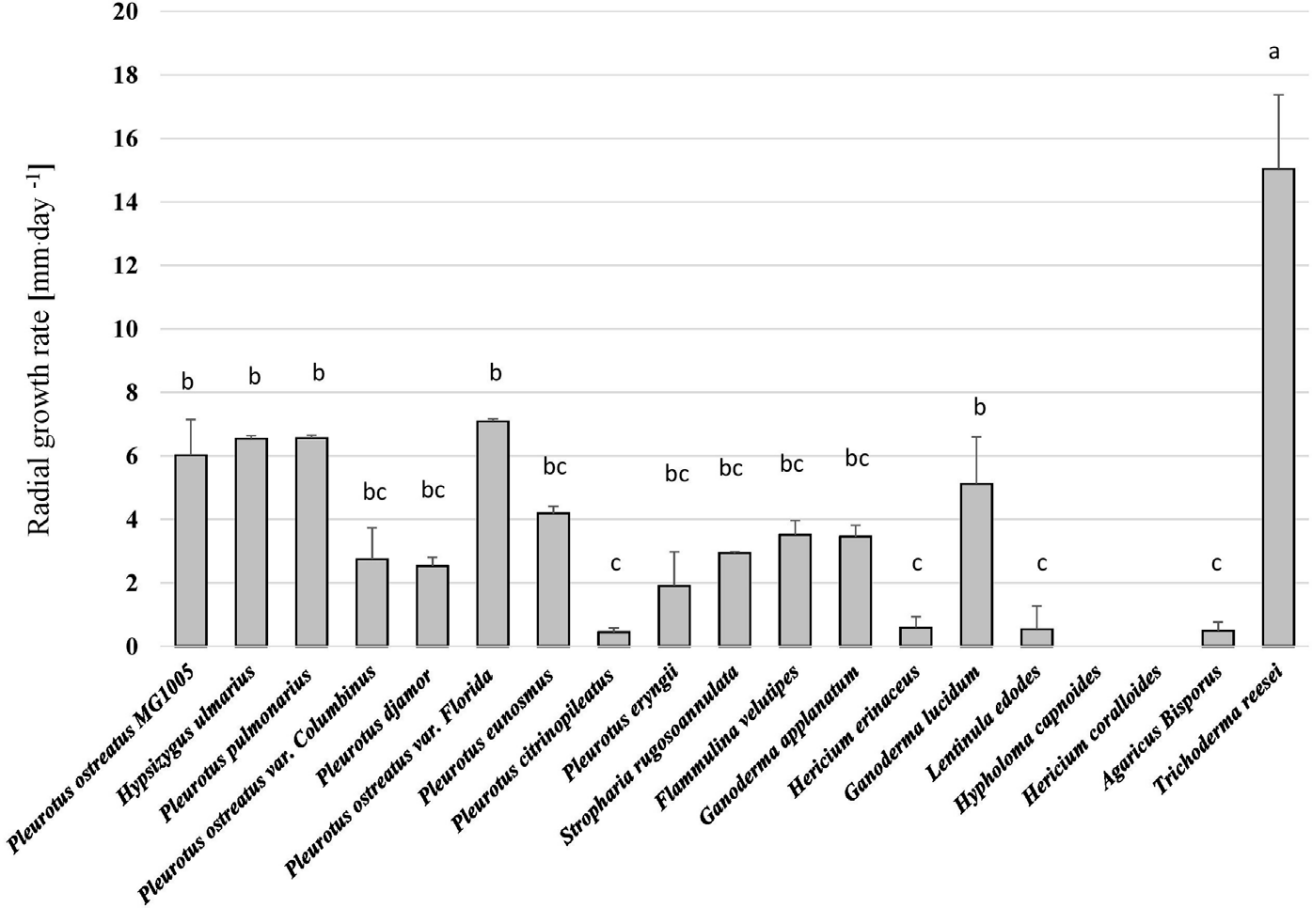
The radial growth rate (mm^.^day^−1^) of different fungi on the hemp waste agar medium. Kruskal–Wallis test followed by Dunn-Bonferroni test (n = 5, *p* < 0.05) revealed significant differences among the treatments and are represented with different letters above the columns.

All fungal strains grew on the hemp-based medium except *H. coralloides* and *H. capnoides* and thus these were excluded from the analysis. Besides the general ability of all other fungal species to grow on the hemp media, significant differences in the growth rates of the fungi were observed (*p* < 0.001, H = 68.72) (Figure 1 and Supplementary Figure S1)*. Trichoderma reesei* (TR), showed the highest growth rate and reached 70 mm of growth during 6 days. *Pleurotus ostreatus* varieties in addition to *G. lucidum* (GL) and *H. ulmarius* (HU) had a lower growth rate, but were significantly better than other tested species. Hence, TR, *P. ostreatus* var*. Florida* (PO), HU, and GL were chosen for the SSF of the hemp waste as they represent diverse genera and sufficient growth on the hemp material.

### 3.2 BSFL feeding experiment

**FIGURE 2:**
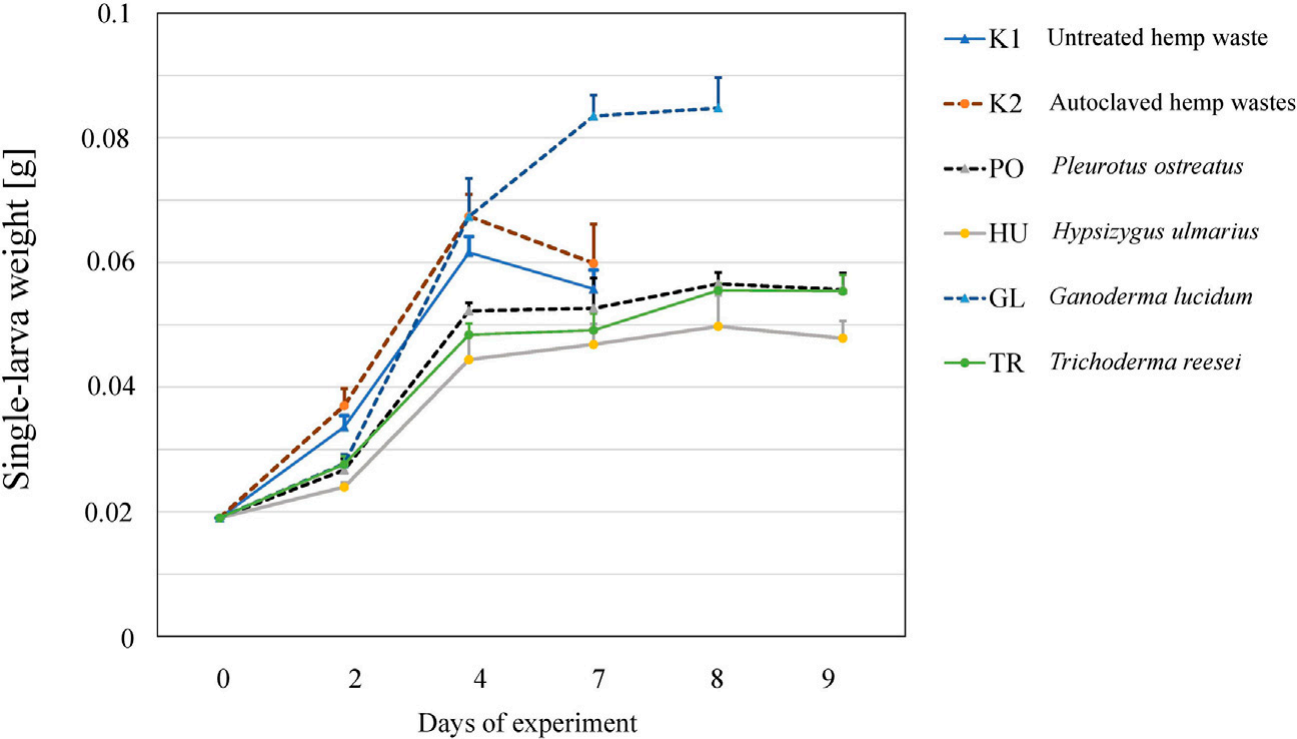
The growth of BSFL on untreated (K1), autoclaved (K2), or fermented hemp waste with different fungal species (PO, HU, GL, and TR). Shown are the mean larval weights and standard deviations over the days of experiments (n = 5).

**FIGURE 3:**
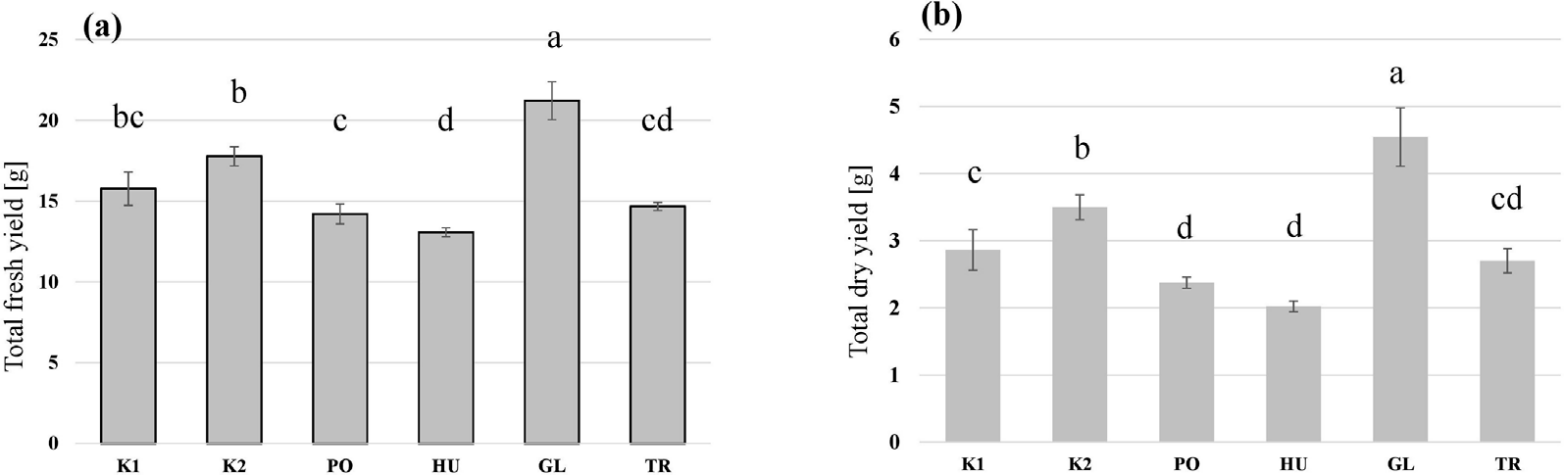
The total fresh yield **(A)** and dry yield **(B)** of black soldier fly larvae (BSFL) provided with untreated hemp-waste (K1), autoclaved hemp-wastes (K2), or hemp wasted fermented with *Pleurotus ostreatus* (PO), *Hypsizygus ulmarius* (HU), *Ganoderma lucidum* (GL), or *Trichoderma reesei* (TR). Shown are the means and standard deviations (n = 5). ANOVA followed by Bonferroni *post hoc* test revealed significant differences among the treatments (*p* < 0.05) which are represented by different letters above the columns.

The larvae grew on all the tested substrates and differences in the growth rate (Figure 2), as well as in the final yield (Figure 3) were observed. The highest growth was observed in the larvae that received GL-fermented substrate, leading to significantly higher fresh yield (F = 83.2, *p* < 0.001 and dry yield (F = 67.7, *p* < 0.001) compared to the rest the treatments (Figure 3). Additionally, the larvae that consumed the autoclaved non-inoculated substrate (K2) had significantly higher growth than the untreated substrate (K1) (Figure 3B). Fermenting the substrate with TR did not influence the final yield compared to the untreated substrate (K1) (Figure 3B), but took more days to reach the growth peak (Figure 2). Fermenting the substrate with PO and HU led to significantly lower dry yields (Figure 3B) compared the control treatments (K1 and K2).

**FIGURE 4:**
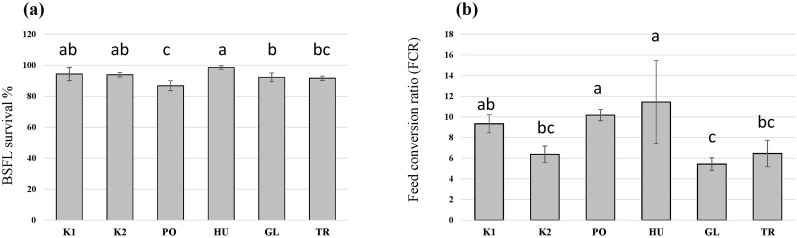
The survival rate **(A)** and the feed conversion ratio (FCR) **(B)** of black soldier fly larvae (BSFL) provided with untreated hemp-waste (K1), autoclaved hemp-wastes (K2), or hemp wasted fermented with *Pleurotus ostreatus* (PO), *Hypsizygus ulmarius* (HU), *Ganoderma lucidum* (GL), or *Trichoderma reesei* (TR). Shown are the means and standard deviations (n = 5). ANOVA followed by Bonferroni *post hoc* test revealed significant differences among the treatments (*p* < 0.05) which are represented by different letters above the columns.

In addition to differences in the growth of BSFL on the different substrates, the survival of BSFL varied among the treatments (F = 10.6, *p* < 0.001) and was decreased in the larvae that received the PO-fermented substrate in comparison to the controls (Figure 4A). Additionally, fermenting the substrate influenced the FCR of the BSFL (F = 8.9, *p* < 0.001), which was the lowest for the autoclaved substrate (K2), and the substrates fermented with GL and TR (Figure 4B). Fermenting the substrate with HU and PO led to an increase in the FCR compared to the autoclaved substrate (K2) without significantly differing from the control untreated hemp waste (K1).

### 3.3 Elemental analysis and crude protein quantification

**FIGURE 5:**
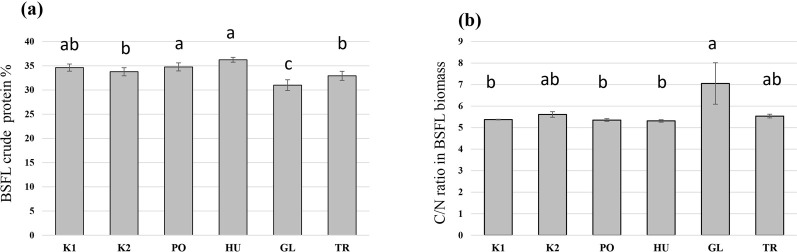
The crude protein values **(A)** and the C/N ratio **(B)** of black soldier fly larvae (BSFL) provided with untreated hemp-waste (K1), autoclaved hemp-wastes (K2), or hemp wasted fermented with *Pleurotus ostreatus* (PO), *Hypsizygus ulmarius* (HU), *Ganoderma lucidum* (GL), or *Trichoderma reesei* (TR). Shown are the means and standard deviations (n = 5). ANOVA followed by Bonferroni *post hoc* test revealed significant differences among the treatments (*p* < 0.05) which are represented by different letters above the columns.

The fermentation of hemp waste significantly influenced the crude protein content of the produced BSFL (F = 21.59, *p* < 0.001). The highest crude protein content was observed in BSFL grown on the PO-fermented and HU-fermented substrates (Figure 5A), both of which differed significantly from the autoclaved control substrate (K2). The crude protein content was significantly lower in the BSFL that received the GL-fermented substrate compared to all other treatments. A reversed trend was observed in the carbon-to-nitrogen (C/N) ratio which was the highest in the BSFL grown on the GL-fermented substrate, and the lowest in the larvae grown on PO- and HU-fermented substrates (Figure 5B). Generally, the produced BSFL had a trend of higher Ca mass fraction compared to the initial hemp waste, and the BSFL produced on untreated hemp wastes (K1) and GL-fermented substrate exhibited a trend of the highest accumulation of Ca (Table 1). A trend of accumulation was also observed in Mn, Zn, P, and S. The values of Cu, Fe, Mg, and K were comparable to those of the initial hemp waste and exhibited a higher or lower trend based on the treatment.

**TABLE 1:** The partial elemental composition of the initial hemp waste substrate and the final larvae produced on the substrate without pre-treatment (K1), after autoclaving (K2), or after fermentation with *Pleurotus ostreatus* (PO), *Hypsizygus ulmarius* (HU), *Ganoderma lucidum* (GL), or *Trichoderma reesei* (TR).

Material	Ca (g/kg)	Cu (mg/kg)	Fe (mg/kg)	K (g/kg)	Mg (g/kg)	Mn (mg/kg)	P (g/kg)	S (g/kg)	Zn (mg/kg)
Hemp Waste	30.36	10.14	312.38	27.73	5.51	299.45	5.09	3.24	53.6
K1	89.83	10.85	376.42	19.48	5.73	821.10	12.15	4.56	136.34
K2	73.96	14.66	267.84	22.49	5.34	714.37	11.03	5.00	165.69
PO	77.51	13.12	251.91	22.17	4.81	769.51	10.67	4.82	159.15
HU	46.63	9.67	237.24	18.18	4.59	845.90	10.07	3.46	125.67
GL	88.55	11.41	204.19	24.01	4.91	849.47	10.27	4.89	104.34
TR	33.15	9.72	368.75	32.36	5.62	316.98	5.49	3.59	58.73

The values based on dry weight. All values are expressed on a dry weight basis.

### 3.4 The fate of hemp secondary metabolites

The initial hemp-waste substrate contained a total of 7,006.5 μg ^.^g^−1^/DW of the analysed cannabinoids, most of which were cannabidiolic acid (CBDA) and cannabidiol (CBD) (2,796.2 μg ^.^g^−1^/DW and 3,413 μg ^.^g^−1^/DW, respectively). The substrate contained 118.4 μg ^.^g^−1^/DW cannabigerolic acid (CBGA), 44.5 μg ^.^g^−1^/DW cannabigerol (CBG), 60.81 μg ^.^g^−1^/DW ∆9-tetrahydrocannabinol (∆^9^-THC), 47.96 μg ^.^g^−1^/DW ∆9-tetrahydrocannabinolic acid (∆^9^-THC-A), 266.36 μg .g^−1^/DW cannabichromene (CBC), and 259.2 μg ^.^g^−1^/DW cannabichromenic acid (CBCA). When the substrate was analysed after autoclaving or SSF with each of the four fungi, the cannabinoid mass fraction was reduced and also differed among the treatments (Table 2). Fermenting the substrate with TR increased the CBD and ∆^9^-THC mass fraction, while reducing the mass fraction of CBDA and ∆^9^-THC-A compared to the initial substrate. In comparison to other fungi, fermenting the substrate with TR lead to a significantly higher mass fraction of CBDA (H = 13.5, *p* = 0.009), CBG (H = 3.8, *p* = 0.05), CBD (H = 11.8, *p* = 0.018), CBC (H = 10.4, p = 0.015), and CBCA (H = 7.26, *p* = 0.027). ∆^9^-THC was only present in the TR-fermented substrate (107.2 μg ^.^g^−1^/DW) and was absent from all other substrate treatments. CBGA and ∆^9^-THC-A were not detected in any of the pre-treated substrates.

**TABLE 2:** The mass fractions of cannabinoids in the initial hemp-waste substrate (K1), the substrate after autoclaving (K2), and in the substrate after solid-state fermentation with *Pleurotus ostreatus* (PO), *Hypsizygus ulmarius* (HU), *Ganoderma lucidum* (GL), or *Trichoderma reesei* (TR), in addition to the concentration of cannabinoids in the black soldier fly larvae (BSFL) biomass produced on the different substrates.

Cannabinoids	Initial waste substrate (K1)	Autoclaved and incubated substrate (K2)	Fermented substrates			
			PO	HU	GL	TR
Cannabidiolic acid (CBDA)	2,796.2	30.7^b^ ±5.4	1.9^c^ ±0.7	0.8^c^ ±0.3	35.2^b^ ±3.8	273.7^a^ ±135.9
Cannabigerolic acid (CBGA)	118.4	-	-	-	-	-
Cannabigerol (CBG)	44.5	26^b^ ±8.7	-	-	-	142.8^a^ ±40.3
Cannabidiol (CBD)	3,413.0	610.2^ab^ ±404.7	9.3^c^ ±3.8	2.8^c^ ±2.5	361.6^b^ ±382.6	5,537.8^a^ ±513.5
∆9-tetrahydrocannabinol (∆^9^-THC)	60.8	-	-	-	-	107.5 ±186.2
Cannabichromene (CBC)	266.4	105.4^b^ ±7.7	2.0^c^ ±3.5	-	78.8^b^ ±43.6	392.2^a^ ±27.9
∆9-tetrahydrocannabinolic acid (∆^9^-THC-A)	48.0	-	-	-	-	-
Cannabichromenic acid (CBCA)	259.2	6.5^ab^ ±1.2	-	-	3.6^b^ ±6.3	121.3^a^ ±40.6
Black soldier fly larvae produced with fermented substrates						
**Cannabinoid**	**K1**	**K2**	**PO**	**HU**	**GL**	**TR**
Cannabidiolic acid (CBDA)	25.9^a^ ±41.9	2.9^b^ ±1.2	-	-	-	12.7^a^ ±4.5
Cannabigerolic acid (CBGA)	-	-	-	-	-	-
Cannabigerol (CBG)	3.3^b^ ±6.0	10.0^b^ ±5.7	-	-	-	36.7^a^ ±5.6
Cannabidiol (CBD)	407.6^b^ ±298.1	485.4^ab^ ±146.5	-	-	96.6^b^ ±19.1	1,586.3^a^ ±206.9
∆9-tetrahydrocannabinol (∆^9^-THC)	6.6^b^ ±14.7	-	-	-	-	58.9^a^ ±7.5
Cannabichromene (CBC)	29.5^b^ ±18.8	37.1^ab^ ±10.9	-	-	15.6^b^ ±3.0	124.1^a^ ±14.1
∆9-tetrahydrocannabinolic acid (∆^9^-THC-A)	-	-	-	-	-	-
Cannabichromenic acid (CBCA)	6.9^b^ ±13.5	0.2^b^ ±0.5	-	-	-	31.6^a^ ±3.3

The mass fractions of cannabinoids were compared between the five different substrate pre-treatments (n = 3) and between the produced BSFL treatments (n = 5). Kruskal–Wallis test followed by Dunn-Bonferroni test (*p* < 0.05) revealed significant differences represented by the different letters. Shown are the mean values in µg.g^−1^/DW ± standard deviations. The significant groups are indicated by different letters.

When the BSFL were fed with the initial hemp waste, compared to the pre-treated variants, the produced insect biomass contained mainly CBD and smaller fractions of other cannabinoids (Table 2). The mass fraction of CBD significantly differed among the treatments (H = 16.14, *p* < 0.001) and was the highest in the BSFL that received the TR-fermented hemp waste. ∆^9^-THC was detected only in the BSFL fed with the untreated hemp waste (K1) (6.5 μg ^.^g^−1^/DW) and the TR-fermented hemp waste (58.9 μg ^.^g^−1^/DW), and the mass fraction significantly differed (H = 13.5, *p* < 0.05). No cannabinoids were detected in the BSFL that grew on the PO- and HU- fermented hemp waste. BSFL that fed on the GL-fermented substrates contained only 96.58 μg ^.^g^−1^/DW and 15.6 μg ^.^g^−1^/DW of CBD and CBC, respectively. The BSFL that received the autoclaved control substrate (K2) had lower CBDA mass fraction compared to the BSFL which received untreated hemp waste substrate (K1) or the TR-fermented substrate (H = 18.3, *p* < 0.001). Additionally, CBG and CBCA values were higher in the BSFL fed with the TR-fermented substrate in comparison to the controls (K1 and K2) (H = 14.8, *p* < 0.05 and H = 15, *p* < 0.05, respectively).

In the untreated hemp waste (K1) different flavonoids from the apigenin and luteolin group could be detected (apigenin, apigenin-glucoronide, apigenin-hexoside-pentoside, three unknown apigenin derivates, luteolin, luteolin-glucoronide). The quantification revealed a total mass fraction of 17.56 μmol ^.^g^−1^, composed of 1.74 μmol ^.^g^−1^ luteolin derivates and 13.82 μmol g^−1^ apigenin derivates (Supplementary Table S2). After substrate pre-treatment, apigenin-glucoronide (3.50 μmol ^.^g^−1^) and luteolin-glucoronide (0.40 μmol ^.^g^−1^) were still detectable in the autoclaved control (K2). In the TR- and PO-fermented substrates only apigenin-glucoronide could be detected (1.75 μmol ^.^g^−1^ DW and PO: 0.10 μmol ^.^g^−1^, respectively). When the produced BSFL were analysed, no hemp-derived flavonoids could be detected.

For terpenes analysis, caryophyllene and neophytadiene could only be detected in trace levels (and thus, not quantifiable) in the untreated hemp waste (K1) and in the substrate fermented with TR. In the autoclaved control substrate (K2) and the GL-fermented substrate, only traces of caryophyllene were detected. All other substrate pre-treatments did not contain any detectable terpenes. Consequently, no terpenes were found in the produced BSFL biomass.

## 4 Discussion

Despite being a multipurpose crop, hemp is still commonly grown for specific purposes resulting in the generation of plant residues with different applications in the bio-economy (Pecoraro et al., 2022; Altman et al., 2023; Yakti et al., 2023a). The hydrolysis of hemp biomass through solid state fermentation can be an approach for the efficient production of sugars and enzymes, enabling the conversion of this waste into valuable products like biofuel (Dessie et al., 2022). Also, fermenting hemp-wastes with fungi such as *P. ostreatus* has been described as an approach to produce enzymes and functional ingredients with various applications in the food and pharmaceutical industry (Setti et al., 2020). The ability of fungi to grow well on this fibre-rich waste can be implemented to enhance its nutritional value for potential use as animal feed.

Recent regulatory changes in several countries have allowed the cultivation of industrial hemp with a THC mass fraction lower than 0.3%, but the use of hemp derived by-product as food or feed is subject to strict regulations (European Commission, 2009). Furthermore, the use of hemp by-products as feed is still a matter of controversy due to possible accumulation of hemp bioactive secondary metabolites in animal products (Wagner et al., 2022). However, to establish recommendations and regulations related to the corresponding use of hemp wastes, it is crucial to generate data on the accumulation of the bioactive phytochemicals in different animal products (Altman et al., 2023) including black soldier fly larvae (BSFL) which have been shown to grow on this waste stream (Yakti et al., 2023a; Leni et al., 2024) Additionally, pre-treatment processes (e.g., fermentation) can potentially enhance the nutritional value of agricultural wastes improving the performance of cultivated BSFL (Peguero et al., 2021). Consistent with this approach, this study aimed to test whether solid-state-fermentation (SSF) pre-treatments of a hemp waste substrate would enhance its nutritional value for the BSFL, and how SSF with different fungi would influence the secondary metabolites composition of the substrate.

In an initial *in vitro* screening, the tested fungal strains varied in their growth on the hemp waste medium (Figure 1, Supplementary Figure S1) and the highest growth rate was observed in *T. reesei* (TR). *Trichoderma* spp. are known for their ability to colonise hemp based materials (Vasiliauskienė et al., 2018), and TR is specifically recognised for its superior metabolic activity and its diverse enzymatic arsenal that enables its growth on lignocellulosic substrates, making it a fungus commonly used in various biotechnological application (Chenthamara et al., 2021), in addition to improving the nutritional value of agricultural wastes (Bulgari et al., 2023). A second well-growing species is *P. ostreatus* (PO), which has been shown to efficiently colonise a hemp spent wastes generated from a cannabinoids extraction processes (Eliopoulos et al., 2022) and degrading hemp stalks lignin via its extracellular enzymatic activity (Xie et al., 2019). In addition to *Pleurotus* spp. and TR, *H. ulmarius* (HU) and *G. lucidum* (GL) exhibited a better growth compared to other tested species. The two fungal species can be cultivated on agricultural and forestry wastes (de Carvalho et al., 2015; Jarial and Bhatia, 2023).

Despite the observed growth of fungi on the hemp waste media, hemp secondary metabolites exhibit antifungal and antibacterial activities (Berardo et al., 2024), which might have persisted in the autoclaved medium hindering or reducing fungal growth. On the other hand, certain microorganisms have been shown to metabolise and degrade hemp secondary metabolites (Rashidi et al., 2009; Ozdemir et al., 2019; Ahmed et al., 2022). A study by Ahmed et al. (2022) demonstrated that fungi, such as *Mucor ramannianus*, *Beauveria bassiana*, and *Absidia glauca,* possess the ability to break down CBD, resulting in diverse metabolites with potential antimicrobial and antiprotozoal activities. In the presented study, fermenting the hemp waste substrate with PO and HU resulted in decreased cannabinoids content compared to the substrate that was only autoclaved and sterile-incubated (K2) (Table 2). On the other hand, a notable decrease in the acidic cannabinoids ∆^9^-THCA and CBDA, and an increase in the more-stable ∆^9^-THC and CBD were observed in the TR-fermented substrate, clearly indicating that decarboxylation took place, which also occurs in heat treatments (Ramirez et al., 2019). This reaction might have conserved the total cannabinoids mass fraction in the substrate and prevented the degradation observed in the sterile-incubated control (K2). The molecular mechanisms of cannabinoids decarboxylation by TR is, however, a subject for future research.

A BSFL feeding trial was carried out to test the influence of SSF treatments on the growth of larvae. SSF can elevate the nutritional value of agricultural residues as lignocellulosic biomass can be transformed into a substrate with improved digestibility (Peguero et al., 2021). In a study by Fitriana et al. (2022) that tested different fungus-waste combinations, SSF improved the waste reduction and nutritional value of cacao pod husk and oil palm fronds for BSFL, leading to higher fatty acid contents in the larvae. Another study by Wong et al. (2021) has shown that the fermentation of coconut endosperm waste with *Rhizopus oligosporus* could also enhance BSFL growth and lead to improved waste reduction. TR has been used to ferment banana peels (Isibika et al., 2019) and trimmings of brassica plants (Lindberg et al., 2022) to improve nutrients availability and consequently improving the BSFL bioconversion process. This, however, was not observed in this study as the TR-fermented hemp waste led to a lower growth rate (Figure 2) and a lower total yield in comparison to the autoclaved control (K2) (Figure 3). Despite the enzymatic activity that could enhance nutrients availability, TR produces antifeedants such as trichocellin A-I and B-II with potential insecticidal activity (Ratnaweera et al., 2021), which might have led to the reduced growth of BSFL feeding on the fermented substrate in our study. This could also explain the reduced growth, and high FCR, of BSFL grown on the HU- and PO-fermented substrates as species of Basidiomycota are capable of producing insecticidal compounds (Sivanandhan et al., 2017). The insecticidal activity of *Pleurotus* extracts have been demonstrated by Rahman et al. (2011) leading to a high mortality rate in the dipteran species *Lucilia cuprina*, which aligns with the findings of the study as higher mortality rates were observed in the BSFL feeding on the PO-fermented substrate (Figure 4).

On the other hand, a superior BSFL growth and yield were observed in BSFL fed with the GL-fermented substrate. A GL isolate has been investigated for its ability to improve the nutritional value of fibre-rich substrates in an SSF setup (Rothmann et al., 2023), and exhibited a remarkable ligninolytic activity while preserving the desired cellulose fraction of the substrate and keeping an excess of available energy in the substrate. This explains the superior growth rate and yield of BSFL on the GL-fermented substrate (Figures 2, 3). The lower crude protein value and the high C/N ratio in the BSFL fed with the GL-fermented substrate suggests that GL boosted BSFL growth by increasing carbon availability in the substrate. This translated to an increase in larval carbon (most likely fat) without a corresponding increase in crude protein (Figure 5).

Numerous factors can influence the elemental composition of BSFL such as the composition of feeding substrate (Newton et al., 2005; Daş et al., 2023), substrate physical structure (Yakti et al., 2023b), and rearing parameters such as the density which could influence nutrient availability and, thus, the composition of BSFL (Yakti et al., 2022). Additionally, the accumulation of elements such as Ca, K, Mg, Na, Cr, Cu, Fe, Mn, Ni, and Zn could vary based on BSF life cycle stage (Rubio et al., 2022). In the presented study, a trend of differences in the partial elemental composition was observed between the different SSF treatments and the controls (Table.1). SSF can increase the biological availability of elements in plant materials and boost the nutritional value (Chawla et al., 2020; Dhull et al., 2021). Furthermore, microorganisms can degrade and utilise antinutrients such as phytic acid (Ayuk et al., 2014; Yakti et al., 2018), consequently enhancing the bioavailability of vital minerals like Fe and Zn, as observed in fermented foods (Mohite et al., 2013). However, an increase trend was only noted in the K values of the BSFL provided with the TR-fermented substrate in comparison to the control, while a trend of lower values was generally observed for other elements. It is known that *Trichoderma* spp. and wood-decaying fungi can alter the accessibility of elements through the release of siderophores and other chelators to form chelate-element complexes (Goodell et al., 1997; Adams et al., 2007). These complexes might have limited the availability of certain elements for the BSFL. However, due to the low BSFL biomass produced in this study, the samples were pooled hindering statistical evaluation of these results. The potential modulation of BSFL composition through substrate fermentation and the mechanisms behind it are a topic for future investigations.

Cannabinoids were found in the produced BSFL and their values differed among the treatments (Table 2). CBD was the most abundant cannabinoid throughout the processes and its occurrence in the produced BSFL appears to correlate with its values in the BSFL substrate. This is in accordance to a previous study that has shown an increase in the cannabinoid content in BSFL with the higher inclusion rate of hemp waste into rearing substrates (Yakti et al., 2023a). Research has explored the impact of cannabinoids such as CBD and THC on the model organism *D. melanogaster*. Cannabinoids exhibited a minimal influence on insect behaviours, yet provided notable neural protective effects following the exposure to traumatic injury (Candib et al., 2024). Despite the absence of the canonical cannabinoid receptor (CB1) in flies (McPartland et al., 2001), cannabinoids could still alter feeding behaviour and metabolism, and alcohol sensitivity of *Drosophila melanogaster* (He et al., 2021a; He et al., 2021b) suggesting alternative pathways and mechanisms for their effects. On the other hand, CBD can exhibit a pesticidal activity against pest insects such as *Manduca sexta* (Park et al., 2019). In the present study no pesticidal activities of cannabinoids could be observed as BSFL survival was not compromised in substrates with higher cannabinoids content (Figure 4), and CBD, as well as other cannabinoids, seemed to be passively absorbed in the BSFL gut as shown in other animals (Cohen and Neuman, 2020).

In terms of BSFL application for feed purposes, enriching the larvae with CBD could potentially elevate the value of the end product. Due to the non-psychoactive properties of CBD and its potential advantages for animal health and welfare, laws concerning its use in animal feed have progressed and numerous countries legalised its use (Fallahi et al., 2022). In cats and dogs, CBD at specific doses could effectively manage stress and range of disorders including osteoarthritis, pruritus, epilepsy, anxiety, and aggression (Corsato Alvarenga et al., 2023; Hunt et al., 2023). CBD also has beneficial effects on the wellbeing of fish by reducing aggressiveness, stress, and cortisol levels (Camargo-dos-Santos et al., 2022). Additionally, chicken could also benefit from CBD, which enhances gut barrier functions and thus preventing potential infections (Konieczka et al., 2020).

Flavonoids and terpenes were almost completely removed by the employed autoclaving process. Metabolite levels in the larvae fell below the detection limits and could no longer be quantified. Chaaban et al. (2017) analysed the thermal stability of different flavonoids, among others luteolin-7-O-glucoside and luteolin. Here, the glycosylated form was found to be more resistant to heat than the aglycon. Nevertheless, a heat treatment of 110°C for 30 min resulted in a significant percentage of loss of around 75% and 93%, respectively. For terpenes, an air-drying temperature of 90°C resulted in a terpene retention in hemp material of 35%–39% (Chen et al., 2021). The results of the authors go hand in hand with the extreme loss of flavonoid contents in our findings. Possible effects of a microbial conversion of flavonoids *via* for example, (de)glycosylation, methylation or glucuronidation, as reviewed in Huynh Nguyen Thai et al. (2014), could unfortunately not be mapped after the autoclaving process due to insufficient mass fraction of target compounds. Corresponding degradation products could not be detected using the applied method.

## 5 Summary and future remark

In this study, solid state fermentation (SSF) using different fungal species was tested on a hemp waste substrate followed by a black soldier fly (BSFL) feeding trial. The fate of cannabinoids and BSFL performance varied based on the fungus employed in the SSF. Autoclaving the substrate without fungus inoculation marginally improved BSFL growth with limited changes in the cannabinoid profile of the produced BSFL. SSF with *T. reesei* (TR) increased CBD and Δ^9^-THC mass fraction in the substrate while decreasing the acidic cannabinoids. Δ^9^-THC was only present in the BSFL that received the untreated and TR-fermented substrate. In terms of BSFL growth, SSF with *G. lucidum* reduced the cannabinoid content and resulted in superior BSFL performance, likely due to enhancing carbon availability in the substrate. *H. ulmarius* and *P. ostreatus* removed the cannabinoids from the substrate and subsequently in the produced larvae. This study confirms that the pre-treatment of hemp waste can be an approach to enhance its nutritional value and remove bioactive secondary metabolites that might otherwise limit its legal utilisation. This research presents preliminary results, and SSF processes parameters (e.g., fermentation time, pH, aeration, nutritional supplements, temperature, *etc.*) can be further optimised leading to potential enhancements of the pre-treatment process. Different fungal species and strains can be utilised in the SSF of hemp waste for the achievement of different biotechnological goals. Future research can focus on exploring the effects of combining different species or strains (consortia) without antagonism but with complementary functions, aiming to harness synergistic benefits. For example, species like GL could enhance larval growth, while PO or HU could contribute to THC degradation.

## Data Availability

The raw data supporting the conclusions of this article will be made available by the authors, without undue reservation.

## References

[B1] Adams P. Lynch J. De Leij F. (2007). Desorption of zinc by extracellularly produced metabolites of Trichoderma harzianum, Trichoderma reesei and Coriolus versicolor. J. Appl. Microbiol. 103 (6), 2240–2247. 10.1111/j.1365-2672.2007.03472.x 18045407

[B2] Addo F. Ominski K. Yang C. Plaizier J. (2023). Quality and safety of hemp meal as a protein supplement for nonlactating dairy cows. J. Dairy Sci. 106, 7602–7612. 10.3168/jds.2023-23222 37641272

[B3] Ahmed S. A. Ibrahim A. K. Radwan M. M. Slade D. Chandra S. Khan I. A. (2022). Microbial biotransformation of cannabidiol (CBD) from cannabis sativa. Planta Medica 88 (05), 389–397. 10.1055/a-1468-3781 33902128

[B4] Altman A. Kent-Dennis C. Klotz J. McLeod K. Vanzant E. Harmon D. (2023). Review: utilizing industrial hemp (Cannabis sativa L.) by-products in livestock rations. Animal Feed Sci. Technol. 307, 115850. 10.1016/j.anifeedsci.2023.115850

[B5] Ayuk A. Iyayi E. Okon B. Ayuk J. Jang E. (2014). Biodegradation of antinutritional factors in whole leaves of Enterolobium cyclocarpum by Aspergillus Niger using solid state fermentation. J. Agric. Sci. 6 (10), 188. 10.5539/jas.v6n10p188

[B6] Beck S. (2022). Detection and quantification of terpenes and terpenoids in different basil species and cultivars. ACS Agric. Sci. and Technol. 2 (5), 988–994. 10.1021/acsagscitech.2c00129

[B7] Berardo M. E. V. Mendieta J. R. Villamonte M. D. Colman S. L. Nercessian D. (2024). Antifungal and antibacterial activities of Cannabis sativa L. resins. J. Ethnopharmacol. 318, 116839. 10.1016/j.jep.2023.116839 37400009

[B8] Bulgari D. Alias C. Peron G. Ribaudo G. Gianoncelli A. Savino S. (2023). Solid-state fermentation of Trichoderma spp.: a New way to valorize the agricultural digestate and produce value-added bioproducts. J. Agric. Food Chem. 71 (9), 3994–4004. 10.1021/acs.jafc.2c07388 36735958 PMC9999421

[B9] Camargo-dos-Santos B. Bellot M. S. Guermandi I. I. Favero-Neto J. da Silva Rodrigues M. da Costa D. F. (2022). Cannabidiol improves fish welfare. Sci. Rep. 12(1):17650. 10.1038/s41598-022-21759-3 36271101 PMC9586945

[B10] Candib A. Lee N. Sam N. Cho E. Rojas J. Hastings R. (2024). The influence of cannabinoids on drosophila behaviors, longevity, and traumatic injury responses of the adult nervous system. Cannabis and Cannabinoid Research 9, e886–e896. 10.1089/can.2022.0285 PMC11295667 37158809

[B11] Carus M. Sarmento L. (2016). The European Hemp Industry: cultivation, processing and applications for fibres, shivs, seeds and flowers. Eur. Ind. Hemp Assoc. 5, 1–9.

[B12] Chaaban H. Ioannou I. Chebil L. Slimane M. Gérardin C. Paris C. (2017). Effect of heat processing on thermal stability and antioxidant activity of six flavonoids. J. Food Process. Preserv. 41 (5), e13203. 10.1111/jfpp.13203

[B13] Chawla P. Kumar V. Bains A. Singh R. Sadh P. K. Kaushik R. (2020). Improvement of mineral absorption and nutritional properties of Citrullus vulgaris seeds using solid-state fermentation. J. Am. Coll. Nutr. 39 (7), 628–635. 10.1080/07315724.2020.1718031 32255407

[B14] Chen C. Wongso I. Putnam D. Khir R. Pan Z. (2021). Effect of hot air and infrared drying on the retention of cannabidiol and terpenes in industrial hemp (Cannabis sativa L.). Industrial Crops Prod. 172, 114051. 10.1016/j.indcrop.2021.114051

[B15] Chenthamara K. Druzhinina I. S. Rahimi M. J. Grujic M. Cai F. (2021). Ecological genomics and evolution of Trichoderma reesei. Trichoderma reesei Methods Protoc. 2234, 1–21. 10.1007/978-1-0716-1048-0_1 33165775

[B16] Cohen L. Neuman M. G. (2020). Cannabis and the gastrointestinal tract. J. Pharm. and Pharm. Sci. 23, 301–313. 10.18433/jpps31242 32762830

[B18] Corsato Alvarenga I. Panickar K. S. Hess H. McGrath S. (2023). Scientific validation of cannabidiol for management of dog and cat diseases. Annu. Rev. Animal Biosci. 11, 227–246. 10.1146/annurev-animal-081122-070236 36790884

[B19] Crini G. Lichtfouse E. Chanet G. Morin-Crini N. (2020). Applications of hemp in textiles, paper industry, insulation and building materials, horticulture, animal nutrition, food and beverages, nutraceuticals, cosmetics and hygiene, medicine, agrochemistry, energy production and environment: a review. Environ. Chem. Lett. 18 (5), 1451–1476. 10.1007/s10311-020-01029-2

[B20] Daş G. Seyedalmoosavi M. Schleifer K. Mielenz M. Metges C. (2023). The validity of the bioaccumulation index versus the bioaccumulation factor for assessment of element accumulation in black soldier fly larvae. J. Insects as Food Feed 9 (10), 1365–1379. 10.3920/jiff2023.0021

[B21] de Carvalho C. S. M. Sales-Campos C. de Carvalho L. P. de Almeida Minhoni M. T. Saad A. L. M. Alquati G. P. (2015). Cultivation and bromatological analysis of the medicinal mushroom Ganoderma lucidum (Curt.: Fr.) P. Karst cultivated in agricultural waste. Afr. J. Biotechnol. 14 (5), 412–418. 10.5897/ajb2014.14022

[B22] Dessie W. Tang J. Wang M. Luo X. Liu X. Qin Z. (2022). One-pot conversion of industrial hemp residue into fermentable feedstocks using green catalyst and enzyme cocktails generated by solid-state fermentation. Industrial Crops Prod. 182, 114885. 10.1016/j.indcrop.2022.114885

[B23] Dhull S. B. Punia S. Kumar R. Kumar M. Nain K. B. Jangra K. (2021). Solid state fermentation of fenugreek (Trigonella foenum-graecum): implications on bioactive compounds, mineral content and *in vitro* bioavailability. J. Food Sci. Technol. 58, 1927–1936. 10.1007/s13197-020-04704-y 33897029 PMC8021647

[B24] Eliopoulos C. Markou G. Chorianopoulos N. Haroutounian S. A. Arapoglou D. (2022). Preliminary research concerning the enrichment of industrial hemp extract residues via solid state fermentation with Pleurotus ostreatus. Appl. Sci. 12 (5), 2376. 10.3390/app12052376

[B25] Ely K. Fike J. (2022). Industrial hemp and hemp byproducts as sustainable feedstuffs in livestock diets. Cannabis/Hemp Sustain. Agric. Mater. 145–162. 10.1007/978-981-16-8778-5_6

[B17] European Commission (2009). Regulation (EC) No 767/2009 of the European parliament and of the council of 13 july 2009 on the placing on the market and use of feed, amending European parliament and council regulation (EC). Off. J. Eur. Union 229, 1–28. No 1831/2003 and repealing Council Directive 79/373/EEC, Commission Directive 80/511/EEC, Council Directives 82/471/EEC, 83/228/EEC, 93/74/EEC, 93/113/EC and 96/25/EC and Commission Decision 2004/217/EC.

[B26] Fallahi S. Bobak Ł. Opaliński S. (2022). Hemp in animal diets—cannabidiol. Animals 12 (19), 2541. 10.3390/ani12192541 36230282 PMC9559627

[B27] Fiani B. Sarhadi K. J. Soula M. Zafar A. Quadri S. A. (2020). Current application of cannabidiol (CBD) in the management and treatment of neurological disorders. Neurol. Sci. 41, 3085–3098. 10.1007/s10072-020-04514-2 32556748

[B28] Fitriana E. L. Jayanegara A. Astuti D. A. Laconi E. B. (2022). Growth performance and nutrient composition of black soldier fly larvae reared on solid-state fermentation substrates with various white rot fungi. Biodiversitas J. Biol. Divers. 23 (9). 10.13057/biodiv/d230959

[B29] Förster N. Dilling S. Ulrichs C. Huyskens-Keil S. (2023). Nutritional diversity in leaves of various amaranth (Amaranthus spp.) genotypes and its resilience to drought stress. J. Appl. Bot. and Food Qual. 96. 10.5073/JABFQ.2023.096.001

[B30] Goodell B. Jellison J. Liu J. Daniel G. Paszczynski A. Fekete F. (1997). Low molecular weight chelators and phenolic compounds isolated from wood decay fungi and their role in the fungal biodegradation of wood1This is paper 2084 of the Maine Agricultural and Forest Experiment Station.1. J. Biotechnol. 53 (2-3), 133–162. 10.1016/s0168-1656(97)01681-7

[B31] Gottlieb S. Food U. Admin D. (2018). Statement from FDA commissioner scott gottlieb. MD, new steps address epidemic youth e-cigarette use.

[B32] Grossule V. Lavagnolo M. C. (2020). Lab tests on semi-aerobic landfilling of MSW under varying conditions of water availability and putrescible waste content. J. Environ. Manag. 256, 109995. 10.1016/j.jenvman.2019.109995 31989971

[B33] He J. Ng S. Y. Tan A. M. X. Yong W. L. Yu F. (2021a). Cannabinoids differentially modulate behavioral and developmental responses to ethanol in Drosophila. bioRxiv 2021, 456340. 10.1101/2021.08.14.456340

[B34] He J. Tan A. M. X. Ng S. Y. Rui M. Yu F. (2021b). Cannabinoids modulate food preference and consumption in *Drosophila melanogaster* . Sci. Rep. 11 (1), 4709–4713. 10.1038/s41598-021-84180-2 33633260 PMC7907270

[B35] Hunt A. B. Flint H. E. Logan D. W. King T. (2023). A single dose of cannabidiol (CBD) positively influences measures of stress in dogs during separation and car travel. Front. Veterinary Sci. 10, 1112604. 10.3389/fvets.2023.1112604 PMC9992179 36908527

[B36] Isibika A. Vinnerås B. Kibazohi O. Zurbrügg C. Lalander C. (2019). Pre-treatment of banana peel to improve composting by black soldier fly (Hermetia illucens (L.), Diptera: stratiomyidae) larvae. Waste Manag. 100, 151–160. 10.1016/j.wasman.2019.09.017 31539755

[B37] Jarial R. Bhatia J. (2023). Performance of various agroforestry wastes for the cultivation of elm oyster mushroom Hypsizygus ulmarius (Agaricomycetes) in India and its biochemical constituents. Int. J. Med. Mushrooms 25 (8), 55–62. 10.1615/intjmedmushrooms.2023049037

[B38] Kee P. E. Cheng Y.-S. Chang J.-S. Yim H. S. Tan J. C. Y. Lam S. S. (2023). Insect biorefinery: a circular economy concept for biowaste conversion to value-added products. Environ. Res. 221, 115284. 10.1016/j.envres.2023.115284 36640934

[B39] Kleinhenz M. D. Magnin G. Ensley S. M. Griffin J. J. Goeser J. Lynch E. (2020). Nutrient concentrations, digestibility, and cannabinoid concentrations of industrial hemp plant components. Appl. Animal Sci. 36 (4), 489–494. 10.15232/aas.2020-02018

[B40] Konieczka P. Szkopek D. Kinsner M. Fotschki B. Juśkiewicz J. Banach J. (2020). Cannabis-derived cannabidiol and nanoselenium improve gut barrier function and affect bacterial enzyme activity in chickens subjected to *C. perfringens* challenge. Veterinary Res. 51, 141–214. 10.1186/s13567-020-00863-0 PMC7682017 33225993

[B41] Leni G. Del Vecchio L. Dellapina C. Moliterni V. M. C. Caligiani A. Cirlini M. (2024). Black soldier fly larvae grown on hemp fiber: nutritional composition and production of potential bioactive peptides. Macromol 4 (1), 135–149. 10.3390/macromol4010007

[B42] Lindberg L. Ermolaev E. Vinnerås B. Lalander C. (2022). Process efficiency and greenhouse gas emissions in black soldier fly larvae composting of fruit and vegetable waste with and without pre-treatment. J. Clean. Prod. 338, 130552. 10.1016/j.jclepro.2022.130552

[B43] Mandrioli M. Tura M. Scotti S. Gallina Toschi T. (2019). Fast detection of 10 cannabinoids by RP-HPLC-UV method in Cannabis sativa L. Molecules 24 (11), 2113. 10.3390/molecules24112113 31167395 PMC6600594

[B44] Marzo C. Díaz A. Caro I. Blandino A. (2019). Valorization of agro-industrial wastes to produce hydrolytic enzymes by fungal solid-state fermentation. Waste Manag. and Res. 37 (2), 149–156. 10.1177/0734242x18798699 30222065

[B45] McPartland J. Di Marzo V. De Petrocellis L. Mercer A. Glass M. (2001). Cannabinoid receptors are absent in insects. J. Comp. Neurology 436 (4), 423–429. 10.1002/cne.1078 11447587

[B46] Mohite B. Chaudhari G. Ingale H. Mahajan V. (2013). Effect of fermentation and processing on *in vitro* mineral estimation of selected fermented foods. Int. Food Res. J. 20 (3), 1373.

[B47] Newton L. Sheppard C. Watson D. W. Burtle G. Dove R. (2005). “Using the black soldier fly, Hermetia illucens, as a value-added tool for the management of swine manure,” in Animal and poultry waste management center (Raleigh, NC: North Carolina State University), 17.

[B48] Ozdemir M. Enisoglu-Atalay V. Bermek H. Ozilhan S. Tarhan N. Catal T. (2019). Removal of a cannabis metabolite from human urine in microbial fuel cells generating electricity. Bioresour. Technol. Rep. 5, 121–126. 10.1016/j.biteb.2019.01.003

[B49] Park S.-H. Staples S. K. Gostin E. L. Smith J. P. Vigil J. J. Seifried D. (2019). Contrasting roles of cannabidiol as an insecticide and rescuing agent for ethanol–induced death in the tobacco hornworm Manduca sexta. Sci. Rep. 9 (1), 10481–10510. 10.1038/s41598-019-47017-7 31324859 PMC6642087

[B50] Pecoraro M. T. Mellinas C. Piccolella S. Garrigos M. C. Pacifico S. (2022). Hemp stem epidermis and cuticle: from waste to starter in bio-based material development. Polymers 14 (14), 2816. 10.3390/polym14142816 35890594 PMC9319283

[B51] Peguero D. A. Gold M. Vandeweyer D. Zurbrügg C. Mathys A. (2021). A review of pretreatment methods to improve agri-food waste bioconversion by black soldier fly larvae. Front. Sustain. Food Syst. 5. 10.3389/fsufs.2021.745894

[B52] Rahman M. F. Karim M. R. Alam M. J. Islam M. F. Habib M. Uddin M. (2011). Insecticidal effect of oyster mushroom (Pleurotus ostreatus) against *Tribolium castaneum* (Herbst). NPAIJ 7 (4), 187–190.

[B53] Raksasat R. Lim J. W. Kiatkittipong W. Kiatkittipong K. Ho Y. C. Lam M. K. (2020). A review of organic waste enrichment for inducing palatability of black soldier fly larvae: wastes to valuable resources. Environ. Pollut. 267, 115488. 10.1016/j.envpol.2020.115488 32891050

[B54] Ramirez C. L. Fanovich M. A. Churio M. S. (2019). Cannabinoids: extraction methods, analysis, and physicochemical characterization. Stud. Nat. Prod. Chem. 61, 143–173. 10.1016/b978-0-444-64183-0.00004-x

[B55] Rashidi H. Akhtar M. T. van der Kooy F. Verpoorte R. Duetz W. A. (2009). Hydroxylation and further oxidation of Δ9-tetrahydrocannabinol by alkane-degrading bacteria. Appl. Environ. Microbiol. 75 (22), 7135–7141. 10.1128/aem.01277-09 19767471 PMC2786519

[B56] Ratnaweera P. B. Madhushika D. H. Jayasundara J. N. M. Williams D. E. de Silva E. D. Andersen R. J. (2021). Antifeedant properties and contact toxicities of the trichocellins AI and B-II from a Trichoderma reesei against Plutella xylostella larvae. Int. J. Trop. Insect Sci. 42, 845–854. 10.1007/s42690-021-00608-2

[B57] Rock E. M. Limebeer C. L. Parker L. A. (2018). Effect of cannabidiolic acid and∆ 9-tetrahydrocannabinol on carrageenan-induced hyperalgesia and edema in a rodent model of inflammatory pain. Psychopharmacology 235 (11), 3259–3271. 10.1007/s00213-018-5034-1 30225659

[B58] Rothmann C. Rothmann L. Viljoen B. Cason E. D. (2023). Application of solid‐state fermentation using mushrooms for the production of animal feed. J. Basic Microbiol. 63 (10), 1153–1164. 10.1002/jobm.202300218 37452386

[B59] Rubio P. Todolí J. Martínez-Sánchez A. Rojo S. (2022). Evolution of the mineral concentration and bioaccumulation of the black soldier fly, Hermetia illucens, feeding on two different larval media. J. Insects as Food Feed 8 (4), 367–378. 10.3920/jiff2021.0068

[B60] Sankari H. S. (2000). Comparison of bast fibre yield and mechanical fibre properties of hemp (Cannabis sativa L.) cultivars. Industrial crops Prod. 11 (1), 73–84. 10.1016/s0926-6690(99)00038-2

[B61] Setti L. Samaei S. P. Maggiore I. Nissen L. Gianotti A. Babini E. (2020). Comparing the effectiveness of three different biorefinery processes at recovering bioactive products from hemp (Cannabis sativa L.) byproduct. Food Bioprocess Technol. 13, 2156–2171. 10.1007/s11947-020-02550-6

[B62] Siddiqui S. A. Ristow B. Rahayu T. Putra N. S. Yuwono N. W. Mategeko B. (2022). Black soldier fly larvae (BSFL) and their affinity for organic waste processing. Waste Manag. 140, 1–13. 10.1016/j.wasman.2021.12.044 35030456

[B63] Sivanandhan S. Khusro A. Paulraj M. G. Ignacimuthu S. Al-Dhabi N. A. (2017). Biocontrol properties of basidiomycetes: an overview. J. Fungi 3 (1), 2. 10.3390/jof3010002 PMC5715959 29371521

[B64] Smets R. Claes J. Van Der Borght M. (2021). On the nitrogen content and a robust nitrogen-to-protein conversion factor of black soldier fly larvae (Hermetia illucens). Anal. Bioanal. Chem. 413 (25), 6365–6377. 10.1007/s00216-021-03595-y 34379169

[B65] Thai H. N. T. Camp J. v. Smagghe G. Raes K. (2014). Improved release and metabolism of flavonoids by steered fermentation processes: a review. Int. J. Mol. Sci. 15 (11), 19369–19388. 10.3390/ijms151119369 25347275 PMC4264116

[B66] Ubando A. T. Felix C. B. Chen W.-H. (2020). Biorefineries in circular bioeconomy: a comprehensive review. Bioresour. Technol. 299, 122585. 10.1016/j.biortech.2019.122585 31901305

[B67] Union E. (2021). Official journal of the European union.

[B68] Vandenberghe L. P. Pandey A. Carvalho J. C. Letti L. A. Woiciechowski A. L. Karp S. G. (2021). Solid-state fermentation technology and innovation for the production of agricultural and animal feed bioproducts. Syst. Microbiol. Biomanufacturing 1, 142–165. 10.1007/s43393-020-00015-7

[B69] Vasiliauskienė D. Balčiūnas G. Urbonavičius J. (2018). “Isolation and identification of fungi growing on fibre hemp shive based thermal insulation materials,” in Proceedings of the 21th Conference for junior researchers “science–Future of Lithuania”, Environmental protection engineering, vilnius, Lithuania, 47–51.

[B70] Wagner B. Gerletti P. Fürst P. Keuth O. Bernsmann T. Martin A. (2022). Transfer of cannabinoids into the milk of dairy cows fed with industrial hemp could lead to Δ9-THC exposure that exceeds acute reference dose. Nat. Food 3 (11), 921–932. 10.1038/s43016-022-00623-7 37118216

[B71] Wong C. Y. Kiatkittipong K. Kiatkittipong W. Ntwampe S. K. Lam M. K. Goh P. S. (2021). Black soldier fly larval valorization benefitting from *ex-situ* fungal fermentation in reducing coconut endosperm waste. Processes 9 (2), 275. 10.3390/pr9020275

[B72] Xie C. Gong W. Zhu Z. Zhou Y. Yan L. Hu Z. (2019). Mapping the secretome and its N-linked glycosylation of Pleurotus eryngii and Pleurotus ostreatus grown on hemp stalks. J. Agric. Food Chem. 67 (19), 5486–5495. 10.1021/acs.jafc.9b00061 31012315

[B73] Xu J. Bai M. Song H. Yang L. Zhu D. Liu H. (2022). Hemp (Cannabis sativa subsp. sativa) Chemical composition and the application of hempseeds in food formulations. Plant Foods Hum. Nutr. 77 (4), 504–513. 10.1007/s11130-022-01013-x 36112300

[B74] Yakti W. Förster N. Müller M. Mewis I. Ulrichs C. (2023a). Hemp waste as a substrate for Hermetia illucens (L.)(Diptera: stratiomyidae) and *Tenebrio molitor* L.(Coleoptera: tenebrionidae) rearing. Insects 14 (2), 183. 10.3390/insects14020183 36835752 PMC9960234

[B75] Yakti W. Kovács G. M. Vági P. Franken P. (2018). Impact of dark septate endophytes on tomato growth and nutrient uptake. Plant Ecol. and Divers. 11 (5-6), 637–648. 10.1080/17550874.2019.1610912

[B76] Yakti W. Müller M. Klost M. Mewis I. Dannehl D. e. Ulrichs C. (2023b). Physical properties of substrates as a driver for Hermetia illucens (L.) (Diptera: stratiomyidae) larvae growth. Humboldt-Universität Berl. 14, 266. 10.3390/insects14030266 PMC10054678 36975951

[B77] Yakti W. Schulz S. Marten V. Mewis I. Padmanabha M. Hempel A.-J. (2022). The effect of rearing scale and density on the growth and nutrient composition of Hermetia illucens (L.)(Diptera: stratiomyidae) larvae. Sustainability 14 (3), 1772. 10.3390/su14031772

[B78] Yakti W. Widjaja E. Förster N. Mewis I. Ulrichs C. (2023c). Evaluating the ability of desert locusts (*Schistocerca gregaria*) to grow when feeding on tomato leaves (Solanum lycopersicum) versus wheatgrass (*Triticum aestivum*). J. Insects as Food Feed 1 (aop), 885–894. 10.1163/23524588-00001027

