# Supplementary material for: Solid-state fermentation of hemp waste: enhancing the performance of Hermetia illucens larvae and altering the composition of hemp secondary metabolites
Source: Front Bioeng Biotechnol. 2025 Jan 24;13:1449233. doi: 10.3389/fbioe.2025.1449233 (PMC11802502; doi:10.3389/fbioe.2025.1449233)
Supplement: Supplementary file 1 [file DataSheet1.docx]

Supplementary Material

## Supplementary figure and table

**
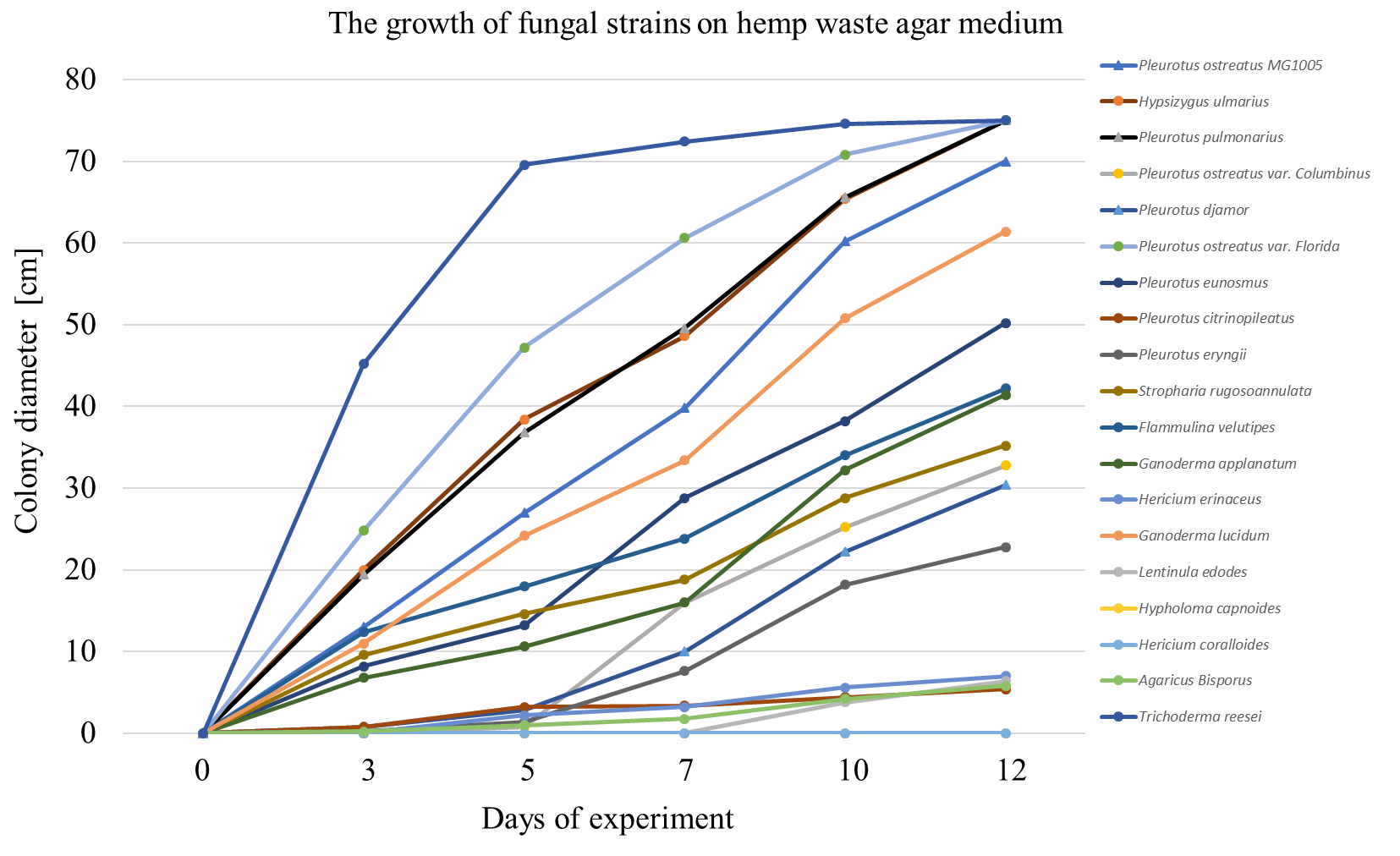
**

**Supplementary Figure S1**: The growth of different fungal strains on the hemp-waste agar medium.

.

**Supplementary Table S1**: Flavonoid content in the initial “untreated" (K1), autoclaved (K2), or the hemp-waste substrate fermented with *Pleurotus ostreatus* (PO), *Hypsizygus ulmarius* (HU), *Ganoderma lucidum* (GL), or *Trichoderma reesei* (TR).

|  | Initial substrate | Substrate after sold state fermentation | | | | |
| --- | --- | --- | --- | --- | --- | --- |
| Flavonoid compound | K1 | TR | GL | HU | PO | K2 |
| Apigenin derivate | 1.26 |  |  |  |  |  |
| Apigenin derivate | 2.50 |  |  |  |  |  |
| Apigenin derivate | 2.18 |  |  |  |  |  |
| Apigenin-hexoside-pentoside | 2.63 |  |  |  |  |  |
| Luteolin-glucoronide | 1.68 |  |  |  |  | 0.40 |
| Apigenin-glucoronide | 6.93 | 1.75 |  |  | 0.10 | 3.50 |
| Luteolin | 0.06 |  |  |  |  |  |
| Apigenin | 0.33 |  |  |  |  |  |
| Total | 17.56 | 1.75 |  |  | 0.10 | 3.89 |
